# Induction of Collagen I by CXCL10 in Ovarian Theca–Stroma Cells *via* the JNK Pathway

**DOI:** 10.3389/fendo.2022.823740

**Published:** 2022-04-01

**Authors:** Chaojun Wang, Yun Sun

**Affiliations:** ^1^ Center for Reproductive Medicine, Renji Hospital, School of Medicine, Shanghai Jiao Tong University, Shanghai, China; ^2^ Shanghai Key Laboratory for Assisted Reproduction and Reproductive Genetics, Shanghai, China

**Keywords:** premature ovarian insufficiency, CXCL10, collagen, luteinized granulosa cells, ovarian theca–stroma cells

## Abstract

Premature ovarian insufficiency (POI) poses a great threat to reproductive-age women. Ovarian fibrogenesis is a basic histologic feature of POI. Ovarian theca–stroma cells are responsible for ovarian fibrosis, but few studies have focused on the ovarian microenvironment. The role and mechanism of chemokines in the development of POI remain unclear. Here, we evaluated C-X-C motif chemokine ligand 10 (CXCL10) in biochemical POI patients, POI patients, and a POI mouse model. CXCL10 levels in serum and follicular fluid were higher in both bPOI and POI patients than in controls. An increased level of CXCL10 was also observed in a POI mouse model. CXCL10 concentrations in serum and follicular fluid were positively associated with follicle-stimulating hormone and negatively associated with antral follicle count. Our study for the first time found that CXCL10 induced COL1A1 and COL1A2 production, two subunits of collagen I in mouse theca–stroma cells by activating the JNK/c-Jun pathway. Inhibition of JNK and c-Jun attenuated the increases of COL1A1 and COL1A2 caused by CXCL10. Moreover, CXCL10 had no effects on hormone synthesis, proliferation, and apoptosis in human luteinized granulosa (hGL) cells. Our findings revealed a potential diagnostic value of CXCL10 in the early stage of POI and shed new insights into the biological function of CXCL10 in ovarian fibrosis.

## Introduction

The prevalence of infertility ranges from 8% to 12% (1). Premature ovarian insufficiency (POI) is a common cause of infertility among reproductive-age women. POI refers to the cessation of ovarian function in women under the age of 40 years. The core features of POI include high follicle-stimulating hormone (FSH) and increased risks of degenerative health problems, including cognitive impairment, cardiovascular disease, and osteoporosis (2). Three stages of the disorder have been demonstrated, namely, occult or subfertility, biochemical POI (bPOI) with a high level of FSH, and overt POI with irregular menstruation (3). It is worth noting that an effective strategy to rescue ovarian function rarely exists in overt POI patients. Therefore, a biomarker that could predict the earlier stage of disease is of great importance.

Ovarian theca–stroma cells differentiate into inner theca cells (TCs) and outer myofibroblast (MFB). By secreting large amounts of extracellular matrix (ECM), MFB is involved in tissue fibrosis (4). Collagen I is the most plentiful component of ECM and widely expressed in the ovary (5, 6). A key characteristic of POI is follicle depletion, which is associated with invasion of the ovarian cortex by ovarian stroma or fibrous tissue (7). Immune abnormalities and inflammation can trigger ovarian fibrosis (7, 8). The role of ovarian theca–stroma cells has mainly been reported in polycystic ovary syndrome (PCOS); however, its role in POI is poorly understood (9, 10).

Accumulating evidence indicates that immune dysregulation is one of the contributing factors of POI (11–13). Circulating autoantibodies and oophoritis have been demonstrated to be present in a subset of POI patients (14, 15). A recent study showed that IFN-γ and TNF-α induced apoptosis and suppressed proliferation by activating NF-κB and JAK/STAT1 signaling in a human granulosa-like tumor cell line and mouse primary granulosa cells. In addition, these two cytokines inhibited estrogen production through CYP19A1 (16). However, whether chemokines are involved in ovarian disturbance remains mostly obscure.

Chemokine (C-X-C motif) ligand 10 (CXCL10), also known as interferon γ-inducible protein (IP-10), is secreted by numerous cell types including endothelial cells, fibroblasts, and macrophages (17, 18). CXCL10 could directly regulate the biology of resident cells by binding to its receptor CXCR3. However, the effect of CXCL10 on ovarian granulosa cells and stromal cells and its underlying mechanism remain poorly defined.

In this study, we comprehensively elucidated for the first time the effects of CXCL10 on disruption of ovarian function in samples from POI patients and in an experimental POI mouse model.

## Materials and Methods

### Recruitment of Patients

A total of 64 women with diverse ovarian reserves, consisting of 23 patients with bPOI, 19 patients with POI, and 22 control women, were enrolled from the Center for Reproductive Medicine, Renji Hospital, School of Medicine, Shanghai Jiao Tong University from May 2019 to June 2020. The research was approved by the Ethics Committee of Renji Hospital with written informed consent from all participants. Women under 40 years of age with an FSH level higher than 25 IU/L on two occasions more than 4 weeks apart are diagnosed as POI. The diagnosis of bPOI was based on FSH level ≥10 IU/L but <25 IU/L and anti-Müllerian hormone (AMH) <1.1 ng/ml. The control group included women who had undergone the first cycle of *in-vitro* fertilization (IVF) or intracytoplasmic sperm injection (ICSI) treatment with tubal or male factor-related infertility. These women aged below 40 years had normal ovarian reserve (FSH < 10 IU/L and AMH ≥ 1.1 ng/ml). They received oocyte retrieval on the same day as the bPOI patients with similar age and BMI. Women with PCOS, hyperthyroidism, hyperprolactinemia, and history of chemotherapy, radiotherapy, or ovarian operation were excluded.

### Sample Collection and Laboratory Tests

Venous blood was obtained from 23 patients with bPOI, 19 patients with POI, and 22 control women on day 3 of the menstrual cycle before their first cycle of IVF or ICSI treatment. The levels of hormones including FSH, luteinizing hormone (LH), progesterone (P_4_), estradiol (E_2_), and AMH were detected by electrochemiluminescence immunoassay kits (Roche, Germany).

The antral follicle count (AFC) of 64 women with diverse ovarian reserves in the early follicular phase was tested through transvaginal ultrasonography.

Follicular fluid was collected from 23 bPOI patients and 22 control women. After ovarian stimulation, women with bPOI and controls underwent oocyte retrieval guided by transvaginal B-scan ultrasound. Ovarian stimulation and oocyte retrieval were based on the conventional protocols of the Center for Reproductive Medicine, Renji Hospital. Follicular fluid from the first large follicle (diameter ≥ 14 mm verified by ultrasound) was collected after the removal of the cumulus complex. Clear follicular fluid without blood contamination was included and centrifuged at 2,500 rpm for 10 min. All samples were stored at −80°C until used. Follicular fluid from the other large follicles of control women undergoing IVF or ICSI was used for primary luteinized granulosa cell isolation.

### Establishment of a POI Mouse Model

All the animals and procedures were approved and supervised by the Institutional Animal Care and Use Committee at Renji Hospital, School of Medicine, Shanghai Jiao Tong University. Female Balb/c mice aged 6–8 weeks (Jiesijie Laboratory Animal, China) were housed under 20°C–25°C temperature and 40%–70% humidity and fed a standard diet. Mice were used in the experiment after acclimatization for 1 week. Mice were divided into the control group (*n* = 10) and experimental group (*n* = 10) randomly. Zona pellucida glycoprotein 3 peptide (ZP3) was purchased from GL Biochem (Shanghai, China) with 95% purity. ZP3 was dissolved and sterilized at 1 mM in double-distilled water, and then emulsified with an equal volume of complete immune adjuvant (CFA, *Mycobacterium tuberculosis* H37RA strain, Sigma, USA). The mice were injected with 0.1 ml of emulsion subcutaneously into the hind footpad after anesthetization with isoflurane. The same method was repeated twice with ZP3 emulsified in incomplete immune adjuvant (IFA, Sigma, USA). The POI mouse model was established 2 weeks later. The mice of the control group were injected with the same volume of saline into the same sites. The four different periods of the estrus cycle (proestrus, estrus, metestrus, and diestrus) were detected *via* vaginal smears. In brief, vaginal secretions were collected at 8 a.m. every day with fine tip pipettes, then stained with methylene blue and observed by microscopy.

### Hematoxylin and Eosin Staining and Follicle Counting

Mice were euthanized at the end of the study and ovaries were collected, which were fixed and used for histopathology examination by hematoxylin and eosin (H&E) staining. Ovary tissues were then sectioned at 5 μm. The follicles with an oocyte containing a clearly visible nucleus were counted. Meanwhile, follicles were divided into primordial, primary, secondary, and atretic ones, as previously described (19, 20). We selected five slides randomly for each group and chose five views on each slide for statistical analysis in a blinded manner.

### Isolation and Culture of Primary Human Luteinized Granulosa Cells

Human luteinized granulosa (hGL) cells were isolated from follicular fluid of control women undergoing IVF or ICSI with tubal or male factor-related infertility as previously described (21). Briefly, cells were obtained by Ficoll-Paque (GE Healthcare, Sweden) after isolation of oocytes and digested with hyaluronidase for 7 min at 37°C. After digestion termination, cells were collected by centrifugation (1,000 rpm, 5 min) and cultured in DMEM/F12 with 10% fetal bovine serum, 100 U/ml penicillin, and 100 mg/ml streptomycin sulfate.

### Isolation and Culture of Ovarian Theca–Stroma Cells

Ovarian theca–stroma cells were obtained from 3- to 4-week-old Balb/c immature mice. In brief, ovaries were isolated from the bursa and poked with insulin needles. After about 100 times, the granulosa cells and oocytes were released. The tissue mixture was digested with pre-equilibrated enzymatic media containing 0.7 mg/ml Collagenase IV (Thermo Fisher Scientific, USA) and 0.2 mg/ml DNAse I (Thermo Fisher Scientific, USA) at 37°C for 1 h. The enzymatic digestion was neutralized with an equal volume of media with 10% FBS and strained by a strainer to remove undigested tissue. Cells were centrifuged (1,000 rpm, 5 min) and suspended in McCoy’s 5A medium with 10% fetal bovine serum and 100 U/ml penicillin. After overnight incubation, the morphological appearance of the theca–stromal cells was observed under a microscope. The attached cells exhibited a long fusiform and fibroblast-like shape. The purity of theca–stromal cells was also identified by immunofluorescence staining of vimentin before further analysis.

### Enzyme-Linked Immunosorbent Assay

Follicular fluid and serum sample of each patient and the serum of each mouse were collected and stored at −80°C for analysis. The expression of CXCL10 in samples from patients and the levels of FSH, estradiol, and CXCL10 in mouse serum were measured using an ELISA kit according to the manufacturer’s instructions.

### Cell Viability Assay

After treatment with different concentrations of CXCL10 for 24 h, the cell viability of hGL cells was quantified by the Cell Counting Kit-8 (CCK-8) assay (ApexBio, China). The absorbance at 450 nm was read on the microplate reader.

### Immunofluorescence Staining

After treatment, hGL and stromal cells in the chamber slide were fixed with 4% paraformaldehyde and then permeabilized with 0.4% Triton X-100. After washing with phosphate-buffered saline (PBS), cells were blocked with 10% bovine serum for 1 h and then incubated with primary antibodies against vimentin (1:100, Santa Cruz, USA) and CXCR3 (1:100, Proteintech, China) overnight at 4°C. After washing with PBS, cells were incubated in the dark for 2 h at room temperature with Alexa Fluor 488 or 598-labeled secondary antibodies (Proteintech, China). The nuclei were stained with DAPI (Servicebio, China). Images were obtained using a microscope with an image analysis system (Zeiss, Germany).

### Masson Staining

Collagen fibers were detected by Masson trichrome staining of ovary tissues from POI mice and control mice. The ovaries of each mouse were harvested and fixed with 4% paraformaldehyde for 24 h. After the paraffin embedding and sectioning process (5 μm in thickness), the ovaries were stained with Masson trichrome. The slides were photographed and analyzed under a light microscope. Collagen fibers were stained blue, muscle fibers and cytoplasm were stained red, and the nuclei were stained black.

### Extraction of RNA for qRT-PCR

Total RNA from cells was isolated using an RNA extraction kit (Foregene, China) and was reverse-transcribed to cDNA with a PrimeScript RT Master Mix Perfect Real-Time kit (TaKaRa, Japan). The target mRNA was quantified with qRT-PCR. The relative mRNA values were normalized to the GAPDH gene control values and calculated using the comparative cycle threshold (ΔΔCt) method.

The primer sequences were as follows: COL1A1 forward 5′-GCTCCTCTTAGGGGCCACT-3′, reverse 5′-CCACGTCTCACCATTGGGG-3′; COL1A2 forward 5′-AAGGGTGCTACTGGACTCCC-3′, reverse 5′-TTGTTACCGGATTCTCCTTTGG-3′; and GAPDH forward 5′-AGGCCGGTGCTGAGTATGTC-3′, reverse 5′-TGCCTGCTTCACCACCTTCT-3′.

### Protein Extraction for Western Blotting

The cells were homogenized in an ice-cold radioimmunoprecipitation assay lysis buffer (CWBio, China) containing a phosphatase inhibitor (Active Motif, USA) and a protease inhibitor (Roche, Switzerland). After quantification of protein concentration by a BCA kit, 40 μg of each sample was separated by 10% or 15% SDS-PAGE gels according to molecular weight, and then transferred onto PVDF membranes. After blocking with 5% BSA for 1 h at room temperature, the blot was incubated with different antibodies against PCNA (1:2,000, Proteintech, China), Bax (1:5,000, Proteintech, China), Bcl2 (1:2,000, Proteintech, China), COL1A1 (1:500, Servicebio, China), COL1A2 (1:2,000, Proteintech, China), JNK (1:3,000, Proteintech, China), phospho-JNK (1:1,000, Proteintech, China), c-Jun (1:1,000, Cell Signaling Technology, USA), phospho-c-Jun (1:1,000, Cell Signaling Technology, USA), tubulin (1:20,000, Proteintech, China), and GAPDH (1:20,000, Proteintech, China). The bands were visualized and captured using a G-Box iChemi Chemiluminescence Image Capture System (Syngene).

### siRNA Transfection

RNAi-negative controls and specific small-interfering RNAs (siRNAs) against c-Jun were purchased from GenePharma (Shanghai, China). To clarify the role of c-Jun in ovarian fibrosis, the ovarian theca–stroma cells from 3- to 4-week-old Balb/c immature mice were transfected with the siRNAs using Lipofectamine 3000 Reagent (Invitrogen, USA) before the different treatments. The c-Jun siRNA sequences are as follows: 5′-GGCACAGCUUAAGCAGAAATT-3′ (sense) and 5′-UUUCUGCUUAAGCUGUGCCTT-3′ (antisense).

### Statistical Analysis

All experimental values are presented as the mean ± SEM. Differences between two groups were analyzed with independent-sample Student’s *t*-tests or Mann–Whitney *U* tests for data with a non-parametric distribution. One-way analysis of variance (ANOVA) followed by Student–Newman–Keuls multiple comparison test was used for comparisons among more than two groups. Spearman’s correlation was applied to evaluate the correlations between CXCL10 levels in serum or follicular fluid and ovarian reserve indicators. *P <*0.05 was considered to be statistically significant.

## Results

### A Higher Level of CXCL10 in Serum and Follicular Fluid of bPOI Patients

In our study, 23 women with bPOI, 19 women with POI, and 22 controls were recruited. The clinical features are shown in **Table 1**. No differences were observed in terms of age, body mass index (BMI), and basal levels of estradiol and progesterone. Compared with control women, both bPOI and POI patients had a higher level of basal FSH and a lower level of AMH (*P* < 0.001). As expected, basal antral follicle counts were decreased in patients with bPOI and patients with POI. A higher level of LH was detected in the POI group than in the bPOI and control groups (*P* < 0.001).

**Table 1:** Demographic features of recruited patients.

Characteristic	Control (*n* = 22)	bPOI (*n* = 23)	POI (*n* = 19)	*P*-value
Age (years)	31.00 ± 0.53	32.22 ± 0.41	30.16 ± 0.96	0.176
BMI (kg/m^2^)	21.89 ± 0.33	22.28 ± 0.53	22.42 ± 0.24	0.407
AFC	14.32 ± 0.62	5.65 ± 0.55^a^	1.58 ± 0.21^a,b^	<0.001
FSH (IU/L)	5.68 ± 0.32	13.59 ± 0.83^a^	37.74 ± 1.88^a,b^	<0.001
LH (IU/L)	4.93 ± 0.37	6.05 ± 0.78	19.76 ± 3.37^a,b^	<0.001
Estradiol (pg/ml)	28.88 ± 2.38	34.09 ± 4.94	25.41 ± 3.72	0.396
P_4_ (ng/ml)	0.23 ± 0.03	0.26 ± 0.03	0.19 ± 0.03	0.783
AMH (ng/ml)	4.01 ± 0.37	0.57 ± 0.06^a^	0.08 ± 0.02^a,b^	<0.001

Hormone levels were measured in serum on day 3 of the menstrual cycle. AFC was detected as the number of follicles (3–8 mm in diameter) by transvaginal ultrasound scan during the early follicular phase.

bPOI, biochemical premature ovarian insufficiency; POI, premature ovarian insufficiency; AFC, antral follicle count; FSH, follicle-stimulating hormone; LH, luteinizing hormone; P_4_, progesterone; AMH, anti-Müllerian hormone.

a P < 0.05 vs. control.

b P < 0.05 vs. bPOI.

**Figure 1:**
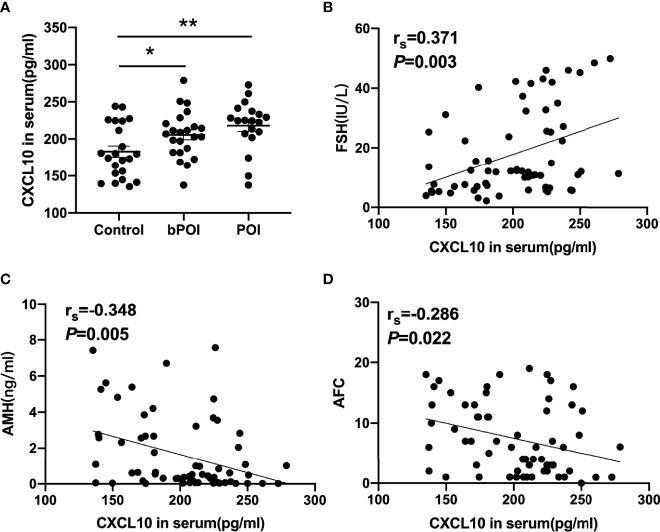
C-X-C motif chemokine ligand 10 (CXCL10) accumulation in the serum of biochemical premature ovarian insufficiency (bPOI) and POI patients. **(A)** CXCL10 levels in the serum from bPOI patients (*n* = 23), POI patients (*n* = 19), and control subjects (*n* = 22). **(B)** Positive correlation between serum CXCL10 levels and follicle-stimulating hormone (FSH) levels. **(C)** Negative correlation between serum CXCL10 levels and AMH levels. **(D)** Negative correlation between serum CXCL10 levels and AFC levels. *P < 0.05, **P < 0.01 vs. control.

**Figure 2:**
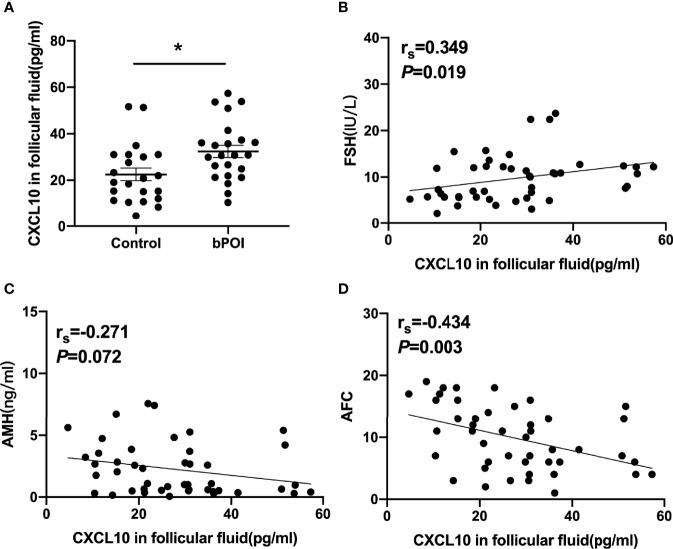
CXCL10 accumulation in follicular fluid of bPOI patients. **(A)** CXCL10 levels in follicular fluid from bPOI patients (*n* = 23) and control subjects (*n* = 22). **(B)** Positive correlation between CXCL10 levels in follicular fluid and serum FSH levels. **(C)** No correlation between CXCL10 levels in follicular fluid and serum AMH levels. **(D)** Negative correlation between CXCL10 levels in follicular fluid and AFC levels. *P < 0.05 vs. control.

The concentrations of CXCL10 in serum and follicular fluid from bPOI patients were significantly increased than those from control women (*P* < 0.05, **Figures 1A**, **2A**). In addition, the level of CXCL10 in serum was higher in POI patients than in control women (*P* < 0.01, **Figure 1A**). As shown in **Figures 1B–D**, CXCL10 plasma levels were positively correlated with FSH, but negatively correlated with AMH and AFC (*P* = 0.003, *P* = 0.005, and *P* = 0.022, respectively).

The correlation of CXCL10 levels in follicular fluid and indicators of the ovarian reserve was analyzed in control women and bPOI patients. The level of CXCL10 in follicular fluid was positively associated with serum FSH (*P* = 0.019, **Figure 2B**) and negatively with AFC (*P* = 0.003, **Figure 2D**), but was unrelated to serum AMH level (*P* = 0.072, **Figure 2C**).

### Abundance of CXCL10 in POI Mice

**Figure 3:**
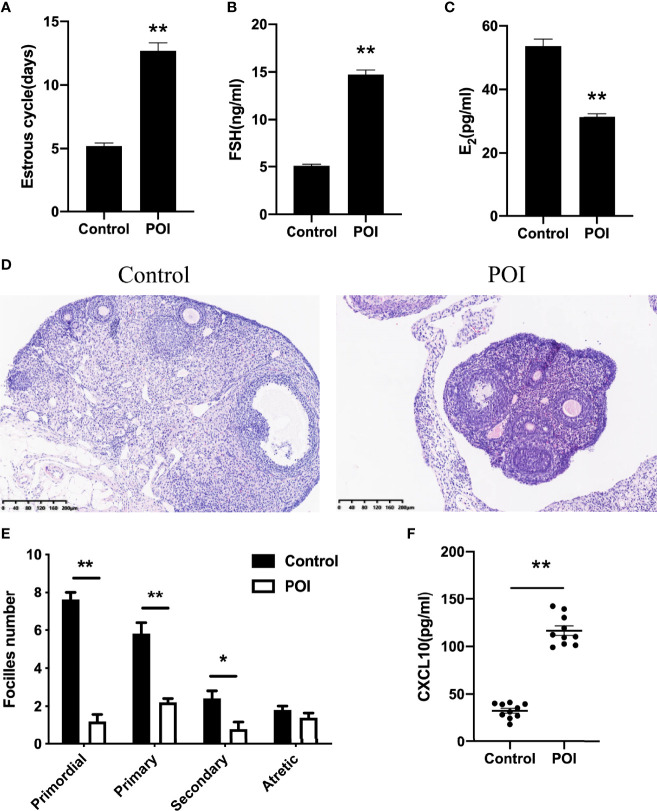
Abundance of CXCL10 in POI mice. **(A)** Estrous cycles of control mice and POI mice. *n* = 10. **(B)** Serum levels of FSH in two groups. *n* = 10. **(C)** Serum levels of estradiol in two groups. *n* = 10. **(D)** Morphological features by H&E staining in two groups. *n* = 10. **(E)** Follicle number changes in the ovaries of two groups. *n* = 5. **(F)** Serum CXCL10 levels in control mice and POI mice. *n* = 10. *P < 0.05, **P < 0.01 vs. control.

To further verify the role of CXCL10 in POI, a ZP3-induced POI mouse model was established. Irregular estrous cycles, elevated plasma FSH level, and declined estradiol level were observed in the POI group compared with the control group (*P* < 0.01, **Figures 3A–C**). Ovarian morphological examination showed that functional follicles, including primordial, primary, and secondary follicles, were significantly reduced in POI mice (*P* < 0.01, *P* < 0.01, and *P* < 0.05, respectively, **Figures 3D, E** **)**. There was no difference in the number of atretic follicles between POI mice and the control group (*P* > 0.05, **Figure 3E**). Compared with control mice, the serum level of CXCL10 was significantly higher in the POI group (*P* < 0.01, **Figure 3F**).

### CXCL10 Had no Effect on Human Luteinized Granulosa Cells

**Figure 4:**
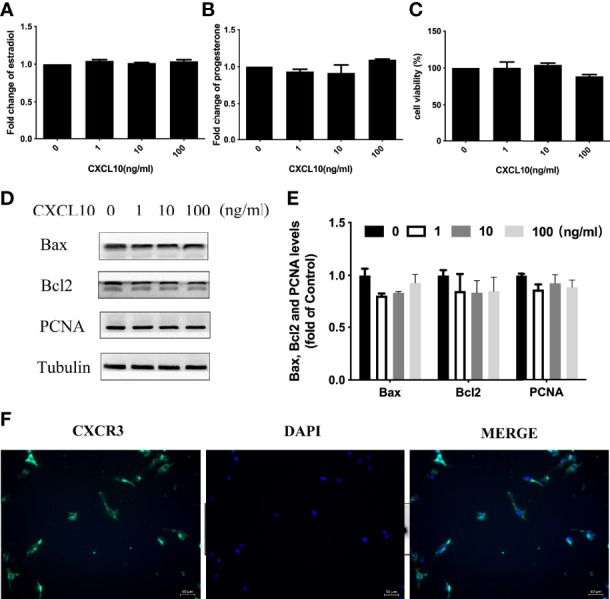
CXCL10 had no effect on human luteinized granulosa (hGL) cells. **(A–E)** The hGL cells were treated with vehicle control (0) and CXCL10 (1, 10, and 100 ng/ml) for 24 h *n* = 3. **(A)** Estradiol levels in culture medium. **(B)** Progesterone levels in culture medium. **(C)** Cell viability of hGL cells. **(D)** Representative Western blot of Bcl2, Bax, and PCNA in hGL cells. **(E)** Quantitative analysis of Bcl2, Bax, and PCNA protein levels. **(F)** Immunofluorescence staining of CXCR3 (green) in granulosa cells.

Both ovarian granulosa cells and theca–stroma cells are indispensable for normal ovarian function. To assess the effects of CXCL10 on ovarian hGL cells, we examined hormone synthesis, cell proliferation, and apoptosis in CXCL10-treated hGL cells. The isolated hGL cells were cultured with different concentrations of CXCL10 for 24 h. As shown in **Figures 4A, B**, CXCL10 had no effect on the production of estradiol and progesterone (*P* > 0.05). Western blot revealed that there were no significant differences in the expression of PCNA, Bcl2, and Bax (*P* > 0.05, **Figures 4D, E** **)**. The CCK-8 assay showed that CXCL10 did not affect cell viability (*P* > 0.05, **Figure 4C**). CXCL10 binds the G protein cognate receptor CXCR3 to exert biological activity. Immunofluorescence staining confirmed the presence of CXCR3 in hGL cells (**Figure 4F**).

### Upregulation of COL1A1 and COL1A2 by CXCL10 in Ovarian Theca–Stroma Cells

**Figure 5:**
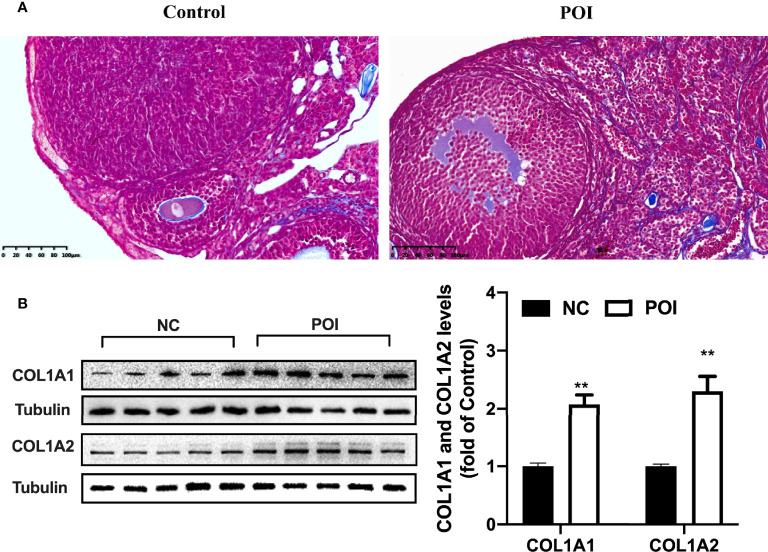
Change of collagen I in the ovaries of POI mice. **(A)** The distribution of blue-staining collagen in the ovaries from control mice and POI mice by Masson staining. **(B)** COL1A1 and COL1A2 protein levels in the ovaries from two groups. n=5. **P < 0.01 vs. control.

**Figure 6:**
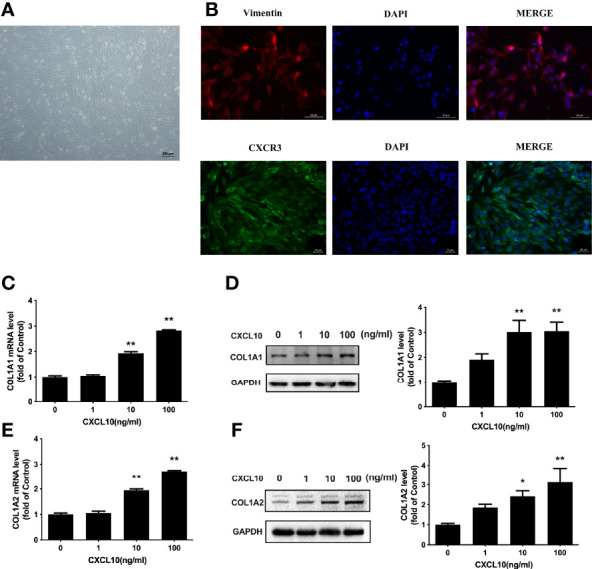
Upregulation of COL1A1 and COL1A2 by CXCL10 in ovarian theca–stroma cells. **(A)** The morphology of ovarian theca–stroma cells under a microscope. **(B)** Immunofluorescence staining of vimentin (red) and CXCR3 (green) in ovarian theca–stroma cells. **(C–F)** Ovarian theca–stroma cells were treated with vehicle control (0) and CXCL10 (1, 10, and 100 ng/ml) for 24 h *n* = 3. **(C)** COL1A1 mRNA level, **(D)** COL1A1 protein level, **(E)** COL1A2 mRNA level, and **(F)** COL1A2 protein level were measured. *P < 0.05, **P < 0.01 vs. vehicle control (0).

Increased collagen abundance was found in the ovarian stroma of POI mice by Masson staining (**Figure 5A**). In addition, Western blot was conducted on collected mice ovarian tissues. COL1A1 and COL1A2 protein levels were increased in the ovaries from POI mice compared with the control mice (*P* < 0.01, **Figure 5B**). To test whether CXCL10 is involved in collagen synthesis during the process of ovarian fibrosis, we incubated primary mouse ovarian theca–stroma cells with different CXCL10 concentrations ranging from 0 to 100 ng/ml for 24 h. The morphology of primary ovarian theca–stroma cells is shown in **Figure 6A** under a microscope. Vimentin, a marker for theca–stroma cells, and CXCR3 were stained positively (**Figure 6B**). *In-vitro* studies demonstrated that CXCL10 upregulated COL1A1 mRNA and protein levels in the concentration of 10 and 100 ng/ml (*P* < 0.01, **Figures 6C, D** **)**. Additionally, the levels of mRNA and protein for COL1A2 were increased by CXCL10 in a dose-dependent manner (**Figures 6E, F** **)**.

### Involvement of JNK and c-Jun in the Regulation of Collagen I by CXCL10

**Figure 7:**
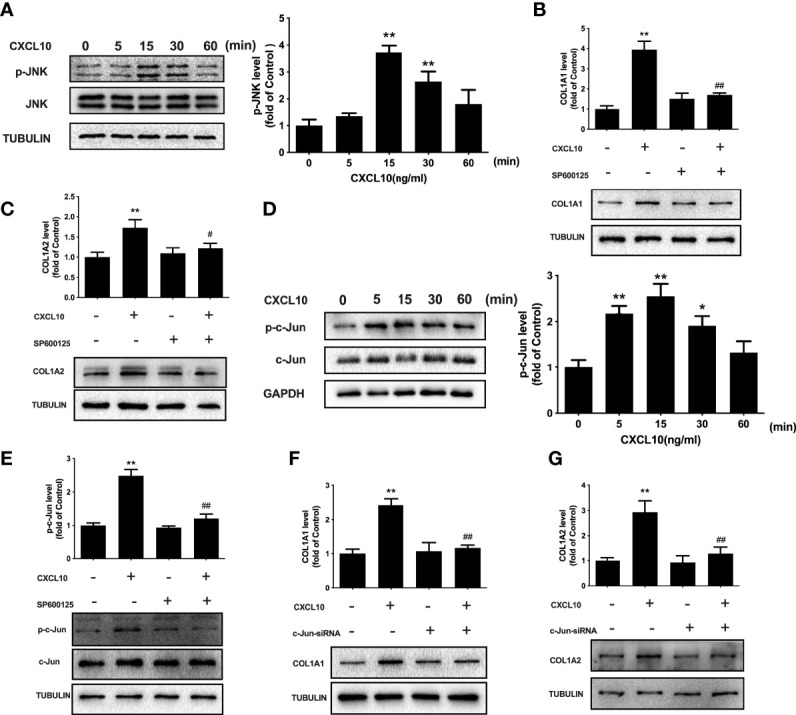
Involvement of JNK and c-Jun in the regulation of collagen I by CXCL10 in ovarian theca–stroma cells. **(A)** Time course effects of CXCL10 (10 ng/ml) on the phosphorylation of JNK in ovarian theca–stroma cells. **(B, C)** Effects of CXCL10 (10 ng/ml, 24 h) on the protein levels of COL1A1 and COL1A2 in the presence or absence of inhibitors for JNK (SP600125, 10 μM). **(D)** Time course effects of CXCL10 (10 ng/ml) on the phosphorylation of c-Jun in ovarian theca–stroma cells. **(E)** Effects of CXCL10 (10 ng/ml, 15 min) on the phosphorylation of c-Jun in the presence or absence of inhibitors for JNK (SP600125, 10 μM). **(F, G)** Effect of CXCL10 (10 ng/ml, 24 h) on COL1A1 and COL1A2 protein levels in the presence or absence of siRNA-mediated knockdown of c-Jun. *n* = 3. *P < 0.05, **P < 0.01 vs. control; ^#^ *P* < 0.05, ^##^ *P* < 0.01 vs. CXCL10.

JNK signaling is related to inflammation and fibrosis. To evaluate the role of JNK signaling in CXCL10-induced stimulation of collagen I, we examined the phosphorylation of JNK after CXCL10 treatment. Time course studies indicated that JNK phosphorylation was markedly increased by CXCL10 at 15 min in ovarian theca–stroma cells (*P* < 0.01, **Figure 7A**). To confirm the involvement of activated JNK in CXCL10-induced collagen synthesis, ovarian theca–stroma cells were treated with CXCL10 in the presence of SP600125 (a JNK inhibitor). SP600125 remarkably inhibited the upregulation of COL1A1 and COL1A2 by CXCL10 (**Figures 7B, C** **)**. The level of c-Jun phosphorylation peaked at 15 min after treatment with 10 ng/ml CXCL10 in ovarian theca–stroma cells (*P* < 0.01, **Figure 7D**). The phosphorylation of c-Jun was inhibited by the JNK inhibitor SP600125 (*P* < 0.01, **Figure 7E**). Furthermore, to understand whether c-Jun was required for CXCL10-mediated induction of collagen I, we transfected ovarian theca–stroma cells with c-Jun siRNA. Western blotting showed that the upregulation of COL1A1 and COL1A2 caused by CXCL10 was abolished by knockdown of c-Jun (*P* < 0.01, **Figures 7F, G** **)**.

## Discussion

In this study, we found that serum CXCL10 levels were increased in women with bPOI and overt POI compared with women with normal ovarian function. Furthermore, a negative correlation between AFC and CXCL10 and a positive correlation between FSH and CXCL10 levels in serum and follicular fluid were found in our study. Our findings demonstrated a potential diagnostic value of a high level of CXCL10 in bPOI and POI patients, suggesting that CXCL10 might be associated with the pathogenesis of POI.

The etiologies of POI include iatrogenic factors, genetic aberrations, and metabolic diseases (22). Nevertheless, the majority of POI cases are idiopathic and their causes remain obscure. Currently, there has been much interest in the potential role of chronic inflammation in POI (23). CXCL10 has been found to be elevated in bPOI patients by a recent study (24), which is in line with our result. However, the role and mechanism of CXCL10 in the pathogenesis of POI have never been reported. We for the first time found that CXCL10 led to collagen synthesis in ovarian theca–stroma cells through the JNK pathway. These findings provide preliminary evidence that CXCL10 played an important role in the pathogenesis of POI.

The development and quality of oocytes depend on a complex ovarian stromal milieu (6). Collagen I was the most widely expressed ECM element within ovaries (25). Therefore, it is critical to evaluate the effect of CXCL10 on collagen I expression in ovarian theca–stroma cells for better understanding the loss of ovarian function. As a major member of the ECM family, we discovered that both COL1A1 and COL1A2 mRNA and protein levels were increased by CXCL10, suggesting that CXCL10 might be associated with the development of POI by promoting collagen synthesis and eventually causing ovarian fibrosis.

It is recognized that CXCL10 has quite opposite effects on tissue fibrosis in different organs. CXCL10-deficient mice exhibited less CCl4-induced liver fibrosis than wild-type mice and a neutralizing anti-CXCL10 antibody significantly inhibited hepatic fibrosis (26). Similarly, Berres et al. (27) found a close link of serum CXCL10 with the progression of liver fibrosis after liver transplantation. In contrast, CXCL10 has an antifibrotic effect on lung and kidney fibrosis. Administration of recombinant CXCL10 in a bleomycin model of pulmonary fibrosis reversed the fibrotic phenotype by binding to syndecan-4 (28). Consistent with the results of *in-vivo* studies, serum CXCL10 level was markedly higher in patients with idiopathic pulmonary fibrosis than in control subjects (29). Zhang and colleagues claimed that recombinant murine CXCL10 attenuated high glucose and TGF-β-induced collagen synthesis in kidney fibroblasts, and this effect was abrogated by the silencing of CXCR3 (30). However, whether CXCL10 activation plays a beneficial or a pathogenetic role in ovarian tissue has never been reported. In the present study, we observed a profibrotic effect of CXCL10 on ovarian theca–stroma cells for the first time. The dual role of CXCL10 on organ fibrogenesis may depend on a diverse microenvironment and activated downstream molecules or signaling pathway (31).

The JNK pathway is involved in a range of physiological processes. Several stimuli have been reported to activate the JNK pathway, such as cytokines, chemokines, oxidative stress, and profibrotic factors. Upon activation, JNK can phosphorylate c-Jun at the NH2-terminal domain by which JNK induces the transcription of genes related to inflammatory and fibrotic responses (32). In human lung and periodontal ligament fibroblasts, researchers reported that JNK phosphorylated c-Jun and enhanced AP-1 transcriptional activity, thus promoting collagen I transcription at the AP-1 binding site (33, 34). Accordingly, we observed JNK and c-Jun activation by CXCL10 in ovarian theca–stroma cells. Furthermore, the JNK inhibitor and c-Jun silencing both reversed the CXCL10-induced collagen I production. These results indicated that phosphorylation of JNK and c-Jun was essential for the induction of collagen I transcription by CXCL10, and subsequently, targeting the JNK and c-Jun pathway had a therapeutic potential to alleviate ovarian fibrogenesis.

The present study has a few limitations. Firstly, the number of bPOI, POI, and control subjects was relatively small. Secondly, we did not collect follicle fluid from patients with POI. Thirdly, we could not illustrate the source of CXCL10. Whether it originates from peripheral blood or from intrinsic cells of ovaries remains unclear. There are several types of POI mouse models, but to date, a no less severe bPOI animal model has been reported (35). Further *in-vivo* studies are needed to investigate the effect of CXCL10 on ovarian fibrosis by recombinant CXCL10 protein. Moreover, it is important to assess the therapeutic potential of CXCL10 neutralization antibody or CXCR3 silencing in both bPOI and POI mouse models.

## Data Availability Statement

The raw data supporting the conclusions of this article will be made available by the authors, without undue reservation.

## Ethics Statement

The studies involving human participants were reviewed and approved by the Ethics Committee of Renji Hospital, School of Medicine, Shanghai Jiao Tong University. The patients/participants provided their written informed consent to participate in this study. The animal study was reviewed and approved by the Institutional Animal Care and Use Committee at Renji Hospital, School of Medicine, Shanghai Jiao Tong University.

## Author Contributions

YS conceived and designed the experiments. CW performed the experiments, analyzed the data, and wrote the paper. All authors contributed to the article and approved the submitted version.

## Funding

This study was supported by the National Key R&D Program of China (2019YFA0802604); National Natural Science Foundation of China (Nos. 82130046, 81771648, 81801408); Shanghai Leading Talent Program, Innovative Research Team of High-Level Local Universities in Shanghai (No. SSMU-ZLCX20180401); Clinical Research Plan of SHDC (SHDC2020CR1046B); Shanghai Municipal Education Commission-Gaofeng Clinical Medicine Grant Support (No. 20161413); and Shanghai Commission of Science and Technology (No. 17DZ2271100).

## Conflict of Interest

The authors declare that the research was conducted in the absence of any commercial or financial relationships that could be construed as a potential conflict of interest.

## Publisher’s Note

All claims expressed in this article are solely those of the authors and do not necessarily represent those of their affiliated organizations, or those of the publisher, the editors and the reviewers. Any product that may be evaluated in this article, or claim that may be made by its manufacturer, is not guaranteed or endorsed by the publisher.
